# A canine model to evaluate the effect of exercise intensity and duration on olfactory detection limits: the running nose

**DOI:** 10.3389/falgy.2024.1367669

**Published:** 2024-05-09

**Authors:** Edgar Aviles-Rosa, Jöerg Schultz, Michele N. Maughan, Jenna D. Gadberry, Dana M. DiPasquale, Brian Farr, Andrea Henderson, Eric Best, Dakota R. Discepolo, Patricia Buckley, Erin B. Perry, Debra L. Zoran, Nathaniel J. Hall

**Affiliations:** ^1^ Department of Animal and Food Science, Texas Tech University, Lubbock, TX, United States; ^2^ Tier Wohl Team GbR, Rödelsee, Germany; ^3^ Excet, Inc., Lexington Park, MD, United States; ^4^ K9Sensus Foundation, Lucas, IA, United States; ^5^ Valiant Harbor International LLC, Potomac, MD, United States; ^6^ Department of Defense Military Working Dog Veterinary Service, San Antonio, TX, United States; ^7^ Department of Emergency Management and Homeland Security, University at Albany, Albany, NY, United States; ^8^ U.S. Army Combat Capabilities Development Command Chemical Biological Center, Edgewood, MD, United States; ^9^ Oak Ridge Institute for Science and Education, US Department of Energy, Oak Ridge, TN, United States; ^10^ Southern Illinois University Carbondale, Carbondale, IL, United States; ^11^ Department of Small Animal Clinical Sciences, Texas A&M University, College Station, TX, United States

**Keywords:** olfactory threshold, detection dogs, exercise duration, signal detection theory, detection performance, exercise intensity

## Abstract

Detection canines serve critical roles to support the military, homeland security and border protection. Some explosive detection tasks are physically demanding for dogs, and prior research suggests this can lead to a reduction in olfactory detection sensitivity. To further evaluate the effect of exercise intensity on olfactory sensitivity, we developed a novel olfactory paradigm that allowed us to measure olfactory detection thresholds while dogs exercised on a treadmill at two different exercise intensities. Dogs (*n* = 3) showed a decrement in olfactory detection for 1-bromooctane at 10^−3^ (v/v) dilutions and lower under greater exercise intensity. Dogs' hit rate for the lowest concentration dropped from 0.87 ± 0.04 when walking at low intensity to below 0.45 ± 0.06 when trotting at moderate intensity. This decline had an interaction with the duration of the session in moderate intensity exercise, whereby dogs performed near 100% detection in the first 10 min of the 8 km/h session, but showed 0% detection after 20 min. Hit rates for high odor concentrations (10^−2^) were relatively stable at both low (1 ± 0.00) and moderate (0.91 ± 0.04) exercise intensities. The paradigm and apparatus developed here may be useful to help further understand causes of operationally relevant olfactory detection threshold decline in dogs.

## Introduction

Working dogs have important detection roles for the military, homeland security, border protection, missing persons, and the biosecurity of food and fiber (1–3). Within the armed forces, dogs remain the primary tool for in-the-field detection of concealed explosives. Dogs primarily detect these threats through olfaction (i.e., their sense of smell), which enables them to identify trace volatiles released from explosive substances (4).

Within the military, some detection tasks are inherently more physically demanding than other non-military detection tasks. An example of a detection task in the military is a dog searching in an open area or clearing a path for military personnel to safely traverse. Although the effect of exercise intensity on olfactory performance has not been extensively studied, existing research suggests that increasing the physical demands of a task can lead to a decrement in detection performance. For instance, several studies have found that exercising dogs on a treadmill before performing an olfactory task led to lower detection performance (5–7).

There are three important limitations of prior research. First, the olfactory tasks were conducted after dogs exercised and not during ongoing exercise. This exercise-stop-detection task approach makes it challenging to evaluate how detection performance changes with intensity and duration of exercise. Developing an exercise-dose-response relationship would be an important metric for detection dogs on patrol required to perform detection tasks during ongoing physical activity. Second, prior research did not evaluate detection thresholds, meaning they did not take into account that low odor concentration can be more challenging to detect than high odor concentration. It is possible that higher physical demands (e.g., exercise) may impact these ends of the spectrum differently. Third, previous studies did not evaluate the effect of speed or gait on detection performance as two studies used a single speed consistent with a trot (5, 7), and one did not report the speed or gait (6).

A paradigm that allows us to measure odor detection threshold changes during ongoing exercise would be a better representation of some types of detection dogs' working conditions. One potential barrier to such research is that there are few available tools to standardize assessment of canine detection capabilities. Our team has recently developed such tools for olfactory assessment including olfactometers designed for canine use (8–10). Although capable of presenting standardized and verifiable odor concentrations while automatically scoring dogs' responses, this olfactory tool has yet to be applied to measure olfaction during exercise—a pivotal element for quantifying the influence of physical activity in olfaction.

The objective of this study was to couple our existing olfactometer technology with a treadmill to develop an olfactory paradigm that allows us to measure the effect of exercise intensity on olfactory detection thresholds. The goal is to develop a laboratory model that is more representative of some detection dog working conditions, particularly in the military.

## Materials and methods

### Participants

The study was conducted at Texas Tech University Canine Olfaction Research and Education Lab. All procedures were approved by the Institutional Animal Care and Use Committee (protocol # 21051-07 & #78018-ST-H.e001).

The sample size consisted of three privately owned dogs that were all three years old (Dasty, Charles and Ziggy). Dogs were brought by their owner for training/testing based on owner availability. Two dogs were mixed breed dogs and one (Dasty) was a Labrador retriever. All dogs were of medium size (18–22 kg). Descriptive information about the dogs can be found in the Supplementary Materials.

All three dogs were in good general health (as indicated by their owners) and owners reported their dogs had no history of physical injury or illness. At the time of testing all dogs were up to date with their vaccines and parasite control and were not receiving any medical treatment.

Prior to the study, owners indicated that Dasty and Ziggy did not perform cardiovascular exercise other than going on regular walks with their owner and having play time (e.g., playing fetch). Charles had a consistent history of regular exercise that included sporadic runs with his owner (e.g., 30-min runs once a week) and multiple weekly sessions of strength conditioning exercises [i.e., Penn Vet Working Dog Fit to Work Program (11)].

### Experimental design

**Figure 1:**
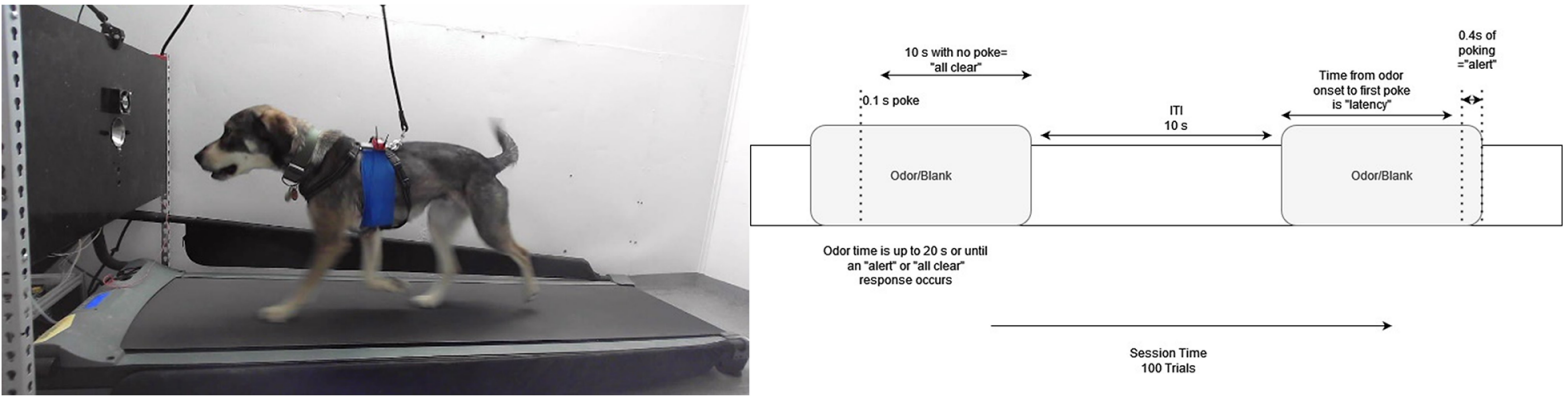
Testing protocol. Left: The odor port is mounted on a panel in front of the dog on top of the treadmill. Safety leash is also attached to the harness. The Polar H10 heart rate monitor was fixed under the self-adherent elastic bandage. The experimenter was located behind the olfactometer panel. Right: Shows the trial parameters. Trials had a set 10 s ITI. A period of 10 s without pokes was considered a no-go “all clear” response. A response of 0.4 s of nose poking was scored as a Go/alert response. Pokes less than the 0.4 s criterion extended the odor on time for an additional 10 s.

To evaluate the effect of exercise intensity and duration on olfactory detection sensitivity, dogs were trained to operate a Go/no-go liquid dilution olfactometer while exercising on a treadmill at either 4 km/h or 8 km/h using a custom designed integrated olfactometer and treadmill. This Olfacto-Treadmill is described in detail below (see Figure 1).

All testing was conducted in a climate-controlled room. The room temperature was set between 20 and 25 °C. Relative humidity ranged from 40% and 50%. Testing was conducted between 8:00 am and 1:00 pm and at least 2 h after meal consumption.

Dogs were trained to walk/trot on the treadmill at 4 and 8 km/h. When a concentration of the target odor (1-bromooctane) was presented as an air stream blown across the length of the treadmill (see odor validation below), the dogs were trained to indicate by inserting their nose (>0.4 s duration) into a stainless-steel port at the front of the treadmill (Go response). If diluent was presented, dogs were trained to not nose-poke and continue exercising (no-go response). All odor presentation was controlled by the computer and the experimenter was always blind to odor status.

Dogs were tested in sessions of 100 Go/no-go trials, which lasted approximately 30 min. Precise session duration was determined by canine performance (time to respond on go trials or false alerts, etc.), but was 28.84 ± 0.64 min. An entire 100 trial session was conducted at one exercise intensity (4 or 8 km/h).

**Figure 2:**
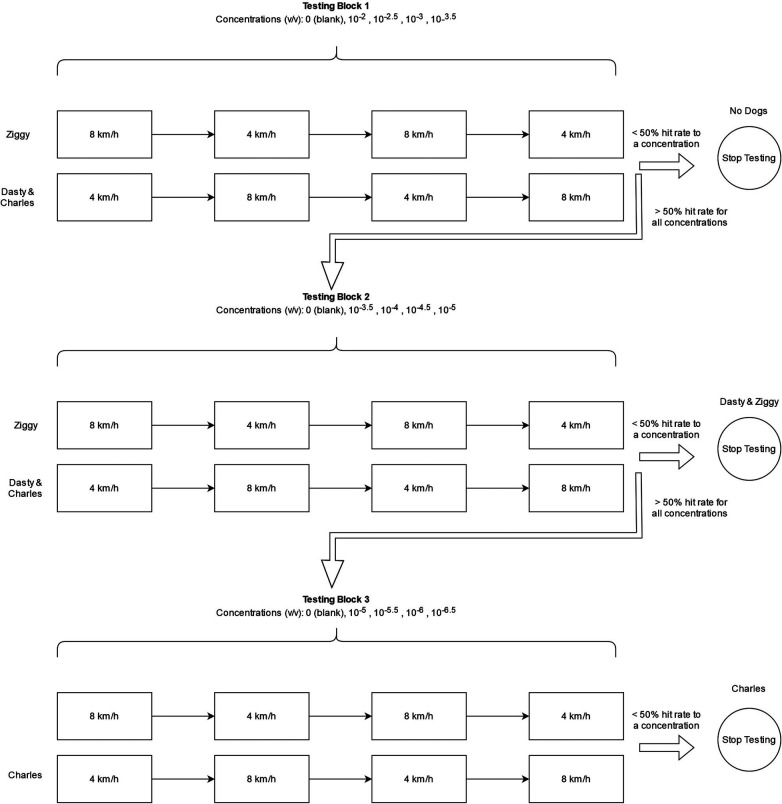
Testing paradigm. Dogs were tested in blocks of concentrations until reaching a 50% hit rate or less for a concentration with either 4 km/h or 8 km/h exercise intensity. Dogs received two sessions of exercise intensity for each block of concentrations before evaluating hit rate in the counterbalanced order as shown. Dog name is shown where each dog met criterion and the order of exercise intensities for that dog. Each block represents 4 100-trial sessions, two at each intensity. These sessions required approximately 30 min. Sessions were separated by a minimum of 2 h and up to 48 h based on owner availability.

To evaluate detection thresholds under each exercise intensity, dogs were trained in blocks of descending odor concentrations (See Figure 2). Each session within a block presented the diluent only (no-go trials) or one of four concentrations of the target odor (described in detail in the detection threshold procedure) in a randomized order. Each block of sessions consisted of four sessions that followed either an 8-4-8-4 or 4-8-4-8 km/h sequence (see Figure 2). Sessions occurred at least 2 h apart and up to 48 h based on owner availability. No more than 2 sessions were conducted per day. 8 km/h sessions were followed by at least 24 h of rest before the next session. Exact spacing between sessions was determined by owner availability.

The first block of concentrations evaluated dilutions from 10^−2^ to 10^−3.5^ in half log steps. Each block evaluated odor concentrations spanning 1.5 log steps. Subsequent Blocks overlapped the lowest dilution to ensure dogs always had one detectable concentration. Thus, Block 2 included the lowest concentration of Block 1 and ranged from 10^−3.5^ to 10^−5^. Block 3 ranged from 10^−5^ to 10^−6.5^ (see Figure 2). After a dog completed a block of sessions, the hit rate (the probability of an alert to an odor present trial) was evaluated for each concentration and exercise intensity. If a dog showed a hit rate <50% for a concentration at either exercise intensity, threshold was considered determined, and testing discontinued. Dasty and Ziggy completed testing after Block 2. Charles completed testing after Block 3.

For all dogs, food was used as the reinforcer. During all sessions, the experimenter was positioned in front of the treadmill, behind the olfactometer panel, and delivered the reinforcer over the olfactometer panel by hand (see Figure 1). We used high value reinforcers such as soft treats, cheese, and hotdogs based on subject preference.

### Integrated olfacto-treadmill apparatus

We coupled an olfactometer with a treadmill (DogTread Pz-1703, Katerno Inc) to test how dogs' olfactory sensitivity was affected by exercise intensity. This setup allowed for olfactory testing while dogs were simultaneously exercising. The treadmill was a purpose built dog treadmill with a standard 2% incline. This incline was used for all training and testing and was not further manipulated.

The olfactometer used was similar to the one described by Aviles-Rosa et al. (10). A detailed description and function of the olfactometer can be found in our previous publication (10). Briefly, the olfactometer was fixed to a wood frame over the treadmill with a polypropylene panel at the front of the treadmill at a height of 55 cm (e.g., dog nose height). The activation/deactivation of the valves was conducted by a computer program. When activated, filtered air from the odor line (2 L/min) entered the odor vial (or a diluent control vial) and carried the headspace of the jar through a Polytetrafluoroethylene (PTFE) tubing that carried the headspace to a PTFE manifold where it was mixed with a continuous air stream (8 L/min). Dogs were trained to respond to the target odor by poking the odor port with their nose. Infrared (IR) sensors were located at the side of the odor port. A nose poke was recorded if the dog broke the IR beam >0.4 s. The use of an olfactometer allowed for complete automation of odor randomization, odor presentation, and data collection enabling double blind trials while the dog was exercising on the treadmill.

#### Odorant

Dogs were trained to 1-bromooctane (CAS # 111-83-1) diluted in food grade mineral oil. This molecule was selected as the target odorant due to its growing usage as a Universal Detector Calibrant (UDC) for detection canines. The initial training concentration was 10% v/v dilution. For threshold assessments, concentration was manipulated through serial dilution ranging from 10^−2^ to 10^−6.5^ v/v with mineral oil.

#### Odor delivery validation

A photoionization detector (PID, 200B miniPID, Aurora Scientific, Canada) was used to validate odor delivery across the span of the treadmill using limonene (CAS: 5989-54-8) diluted in mineral oil (5 × 10^−2^ v/v) as a tracer odorant. A tracer odorant was used due to poor sensitivity of the PID to the target 1-bromooctane. We selected limonene as a tracer due to PID sensitivity, and its relatively similar molecular weight (135 g/mol vs. 193 g/mol) and vapor pressure (1.55 vs. 0.35 mmHg) to 1-bromooctane. The sensor was mounted on a stand at odor port height (what would be nose height of the dogs tested) and placed on the treadmill at distances from 7 cm to 76 cm from the odor port. Odor was presented for 45 s followed by 60 s without odor. Measures were repeated 10 times for each distance. The odor airline and continuous air dilution line were set to identical flows to training and testing (e.g., 2 L/min odor line and 8 L/min continuous line). The order in which the different distances were evaluated was randomized. During the activation/deactivation cycle, voltage readings from the PID were recorded at 500 Hz. The raw voltage readings were filtered with a low-pass Butterworth filter at a scalar of 0.01 of the Nyquist frequency using the signal package in R. To address sensor drift from baseline (i.e., negative voltage values) the average voltage reading when the odor valve was deactivated was calculated and subtracted from the average voltage readings when the odor valve was activated. A positive voltage difference indicated that the sensor was detecting the odorant. A regression analysis was conducted to evaluate the effect of distance on changes in voltage when the odor valve was activated. A calibration curve was conducted by recording the PID response to the headspace of a vial with different liquid dilutions of limonene. Using the calibration curve, we were able to estimate how the odor concentration changed at different locations on the treadmill.

### Olfacto-treadmill training

Dogs were trained to operate the Olfacto-treadmill in a series of training steps described herein. After meeting 80% detection accuracy or higher at the final training step, dogs proceeded to formal testing.

#### Treadmill familiarization

Dogs were initially familiarized with exercise on the treadmill without the olfactometer. We first trained dogs to step up on the treadmill on cue. Within two sessions dogs were readily stepping on the treadmill. Subsequently, while the experimenter was luring the dog with food from the front of the treadmill, we started the treadmill at a walk pace (<4 km/h) for a couple of seconds so the dogs could get used to the belt movement and motor pattern. None of the dogs showed behaviors indicative of stress when the belt was moving. For all dogs, the duration and speed of the walk was increased to 10 min and 4 km/h within 5 sessions. Dogs were gradually acclimated to higher speeds on the treadmill by adding short intervals where the speed was increased to 8 km/h until they were able to exercise at 8 km/h for 20 consecutive minutes. For dogs' safety, the experimenter always had the leash in hand (with no pressure on the dog), and the leash was attached to the harness (PetSafe 3 in 1 harness; Knoxville, TN). Dogs were free to step off the treadmill at any point during any session. The olfactometer was added to the treadmill once dogs completed two 20-min sessions exercising at both 4 and 8 km/h spanning multiple days.

#### Indication of target or go response

The first phase of training consisted of training dogs on the Go response or indication. The Go response or indication consisted of poking the odor port (breaking the IR beam) when the target odor was presented while simultaneously walking or trotting on the treadmill. Dogs had 20-trial sessions with odor presented at each trial while they were walking. A trial consisted of the presentation of odor for 20 s followed by a 10-s intertrial interval (ITI) where the odor valve was deactivated to allow for odor clearance before the next trial. While odor was present, the experimenter reinforced successive approximations to the odor port (i.e., shaping). This was repeated until dogs were triggering the IR sensor in front of the port within the 20 s the odor was present. Responses during the ITI were not reinforced (i.e., extinction). This was repeated until dogs learned to activate the IR sensor by holding the nose in the port for at least 0.4 s while walking.

#### Introduction of distracting odors

Once dogs were triggering the IR sensor consistently at the presentation of the target odor, distractor odors were introduced to train the No-go response. This consisted of not poking the odor port for a predetermined period of time when a distractor odor was presented. A blank vial, mineral oil (MO), Limonene (CAS: 5989-54-8; 10-2 v/v), Hexanal (CAS # 66–25–1; 10-2 v/v), and Isobutyl propionate (CAS # 540-42-1; 10-2 v/v) were used as distracting odors.

Within a 20-trial session, dogs were randomly presented either the target or a distractor odor with a 50% probability each (e.g., 10 trials with the target and 10 trials with distractor odors). Responses to distractor odors (e.g., poking the port) were ignored (extinction) and did not result in the termination of the trial. Not responding to distractors for 3 consecutive seconds was reinforced and resulted in the termination of the trial. The number of trials in a session increased gradually to 60 trials. Additionally, the criterion for not responding to distractors was increased to 8 s. Next, treadmill speed was alternated between 4 and 8 km/h until dogs were able to perform all 60 trials while exercising at a trot for 15–20 min.

#### Variable reinforcement rate and testing parameters

In the final training phase, the number of trials in a session was increased to 100. 50% of trials were target present trials (Go trials). Dogs had 10 s to make a poke or “alert” response, otherwise an “all clear” response was scored. If a dog poked below the 0.4-s threshold during the initial 10-s window, the timer extended, allowing for up to an additional 10 s for a response (see Figure 1). The ITI remained at 10 s. The reinforcement rate for correct rejections (i.e., no indication to distractors) gradually decreased to 40% to maintain high hit rates. Correct rejections to be reinforced were randomized by the computer program. Dogs were always reinforced for correct alerts to the target odor. False alerts (i.e., response to distractors) and false rejections (i.e., no response to target odor) resulted in the termination of the trial with no reinforcement.

At the end of training, the proportion of false alerts and misses were 0.05 ± 0.02 (SE) and 0.02 ± 0.01 (SE), respectively. The proportion of correct responses to 1-Bromooctane for all dogs was above or equal to 90% (e.g., more than 45 correct responses out of 50 trials with target odor).

### Olfactory detection threshold sessions

A testing session consisted of 100 trials (∼28–33 min) at a target odor frequency of 50%. Prior to a testing session dogs walked on the treadmill for two-min as a warm up. Presentation of target and distractor odor was randomized by the computer program. All correct responses to the target odor were reinforced, and 40% of the correct rejections were reinforced. Correct rejections to be reinforced were randomized by the computer program. Trial parameters were the same as at the end of the training phase in which all dogs reached the training criterion. All testing was double blind. Different sounds were used to indicate to the experimenter whether the dog was correct and should be reinforced. False alerts and odor misses were marked with a “buzzer” sound. These responses were not reinforced and resulted in the immediate termination of the trial. Hits and correct rejections preprogrammed to be reinforced were marked with a “bleep” sound which indicated to the experimenter to deliver the reinforcer. Unreinforced correct rejections were not marked with a tone.

Within one session, dogs were presented 4 half-log liquid dilutions of 1-bromooctane or mineral oil (diluent). Fifty trials presented only the diluent odor and 50 trials presented one of the concentrations. Each concentration was presented 12–13 times per session. To ensure that the presentation of all concentrations was distributed equally across a testing session, odor randomization was conducted in sets of 10 trials. That is, within a set of 10 trials the distractor odor was presented in 5 trials (e.g., 50% target odor frequency) and each concentration was presented at least once to the dog. One concentration was tested twice to account for the tenth trial as there were only 4 concentrations. Four concentrations per session were used due to the capacity of the olfactometer used.

#### Heart rate

The dogs' heart rate (HR) was measured during each session, and all dogs were familiarized with the measurement equipment before testing. Heart rate was measured with the Polar® H10 (12, Kempele, Finland) which was attached to the chest with the manufacturer's strap and an additional 10 cm wide self-adherent elastic bandage (Vet Wrap, Marieta GA). Ultrasound gel was applied to ensure electrode connectivity. We used the Actilife software to record heart rate at 1 Hz.

### Statistical analysis

We calculated the hit rate (number of alerts to an odor divided by number of trials that odor was present) and correct rejection rate (number of no alerts divided by number of no odor trials). Additionally, the computer program recorded the latency of a response as the number of seconds a dog required to trigger the IR sensor after the start of odor presentation. For trials in which the dog did not respond, latency was set to 10 s.

Signal detection measures were also calculated to evaluate how dogs' sensitivity and response bias were affected by increased exercise intensity. We utilized non-parametric signal detection theory measurements (12). *A'* (non-parametric measure of sensitivity; analog of *d'*) and *B”* (non-parametric measure of response bias; analog to *c*) were calculated using the equations described by Stanislaw and Todorov (12). *A'* values range from 0 to 1. Values of 1 indicate perfect performance, whereas values lower than 0.5 indicate a lack of discrimination (12). *B”* values range from −1 to 1. Values of zero indicate no response bias, whereas negative and positive values indicate the degree of bias towards the Go and No-go response, respectively (12).

Using timestamps from the Actilife software and the olfactometer program we were able to calculate an average HR per trial. For this we averaged the data obtained from the beginning of a trial until the end of a trial.

A scaled score for odor concentration was also calculated to standardize concentration with respect to an individual dog's detection threshold, which we refer to as “dilution steps”. We divided each concentration by the individual dog's detection threshold (the first concentration with ≤0.5 hit rate) followed by Log_10_ transformation. Thus, if a dog's threshold was at 0.001 v/v dilution, the concentration of 0.00316 would be calculated as Log_10_ (0.00316/0.001) yielding a 0.5 “dilution step” above threshold for that dog. A concentration of 0.01 would therefore be 1 dilution step and 0.316 would be 1.5 dilution steps, etc. This allowed us to account for individual differences in odor detection threshold for analyses. Due to the limited sample size, we did not conduct null hypothesis significance testing.

## Results

### Odor delivery validation

**Figure 3:**
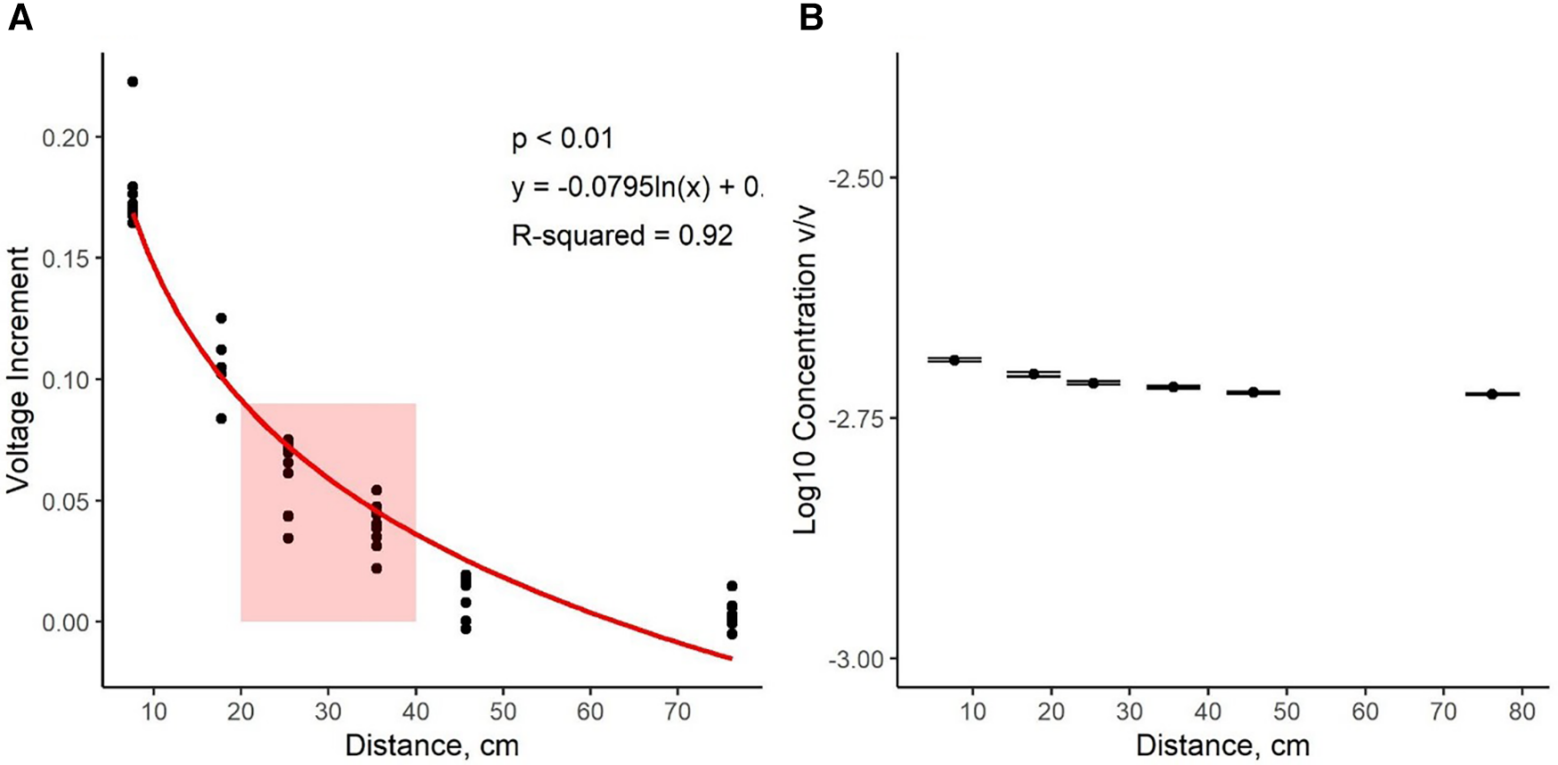
Data from the photo ionization detector (PID). (**A**) shows voltage increments as a dependent variable (*y*-axis) and distance from the odor port (cm) as the independent variable (*x*-axis). The red box illustrates the location of the dogs’ nose on the treadmill. Increasing the distance from the port resulted in lower voltage increments. A logarithmic regression describing the relationship between distance and voltage increment was statistically significant. (**B**) shows that the estimated concentration of limonene was similar at all the distance tested.

Figure 3 shows the average voltage increment measured by the PID at each distance tested. Limonene volatiles were detected at distances of up to 76.2 cm from the odor port quickly after the activation of the odor valve. As the distance decreased, the PID voltage exhibited a logarithmic increase (*P* < 0.001; *R*^2^ = 0.92 with the linear regression model $y\;=\;-0.0795\log_{10}(x)\;+\;0.255$). Nonetheless, Figure 3B shows how the estimated concentration of limonene was relatively constant (within a half-log step) at all the difference distance tested (0.002 ± 7.67 × 10^−6^). A red box displays the range of where the dog's nose would most likely be located during odor presentation based on the length of the treadmill.

### Heart rate

**Figure 4:**
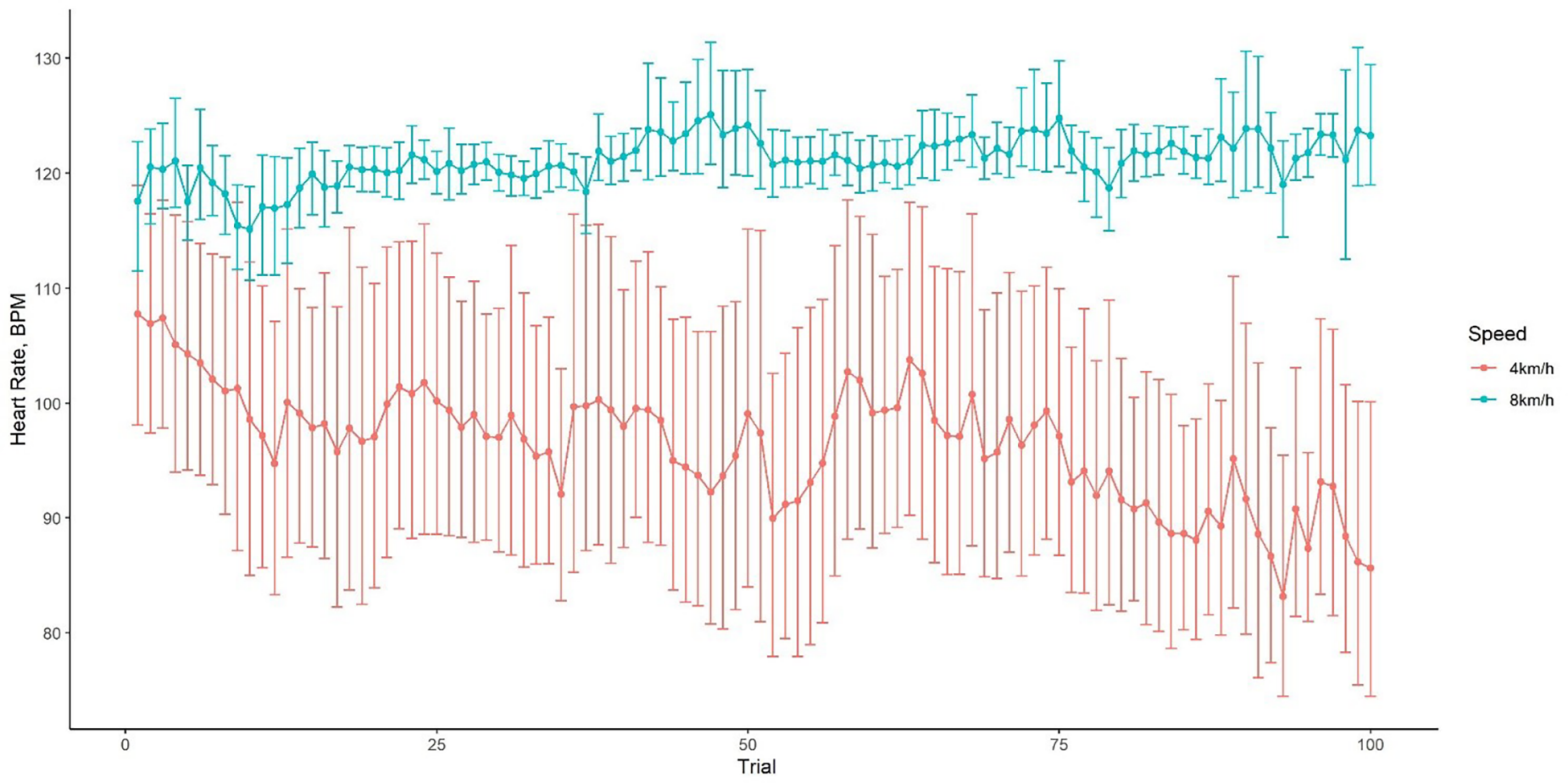
Mean heart rate of study participants ±95% confidence intervals within a session exercising at 4 km/h (low intensity) and 8 km/h (moderate intensity).

HR did not increase with trial and, as expected for steady state exercise, remained relatively constant throughout a session (see Figure 4). The HR at 4 km/h was 95.9 ± 21.6 (mean ± SD) bpm whereas it was 121.2 ± 6.6 bpm at 8 km/h. These HR values correspond to low and moderate exercise intensities for dogs based on previous literature in which canine maximal heart rates exceed 300 bpm (13, 14). We therefore refer to 4 km/h as “low” intensity and 8 km/h as “moderate” intensity. Dasty's and Ziggy's average HR at a walk were 86.55 ± 14.09 bpm and 88.01 ± 16.60 bpm, respectively. Charles' average HR at a walk was 117.83 ± 19.47 bpm. Despite HR differences between Charles and the other dogs at a walk, all dogs had a similar HR at a trot. At a trot, Dasty showed the highest average HR (125.29 ± 3.05 bpm). Ziggy and Charles HR were 119.66 ± 7.32 bpm and 119.12 ± 6.67 bpm, respectively.

### Olfactory detection threshold

**Figure 5:**
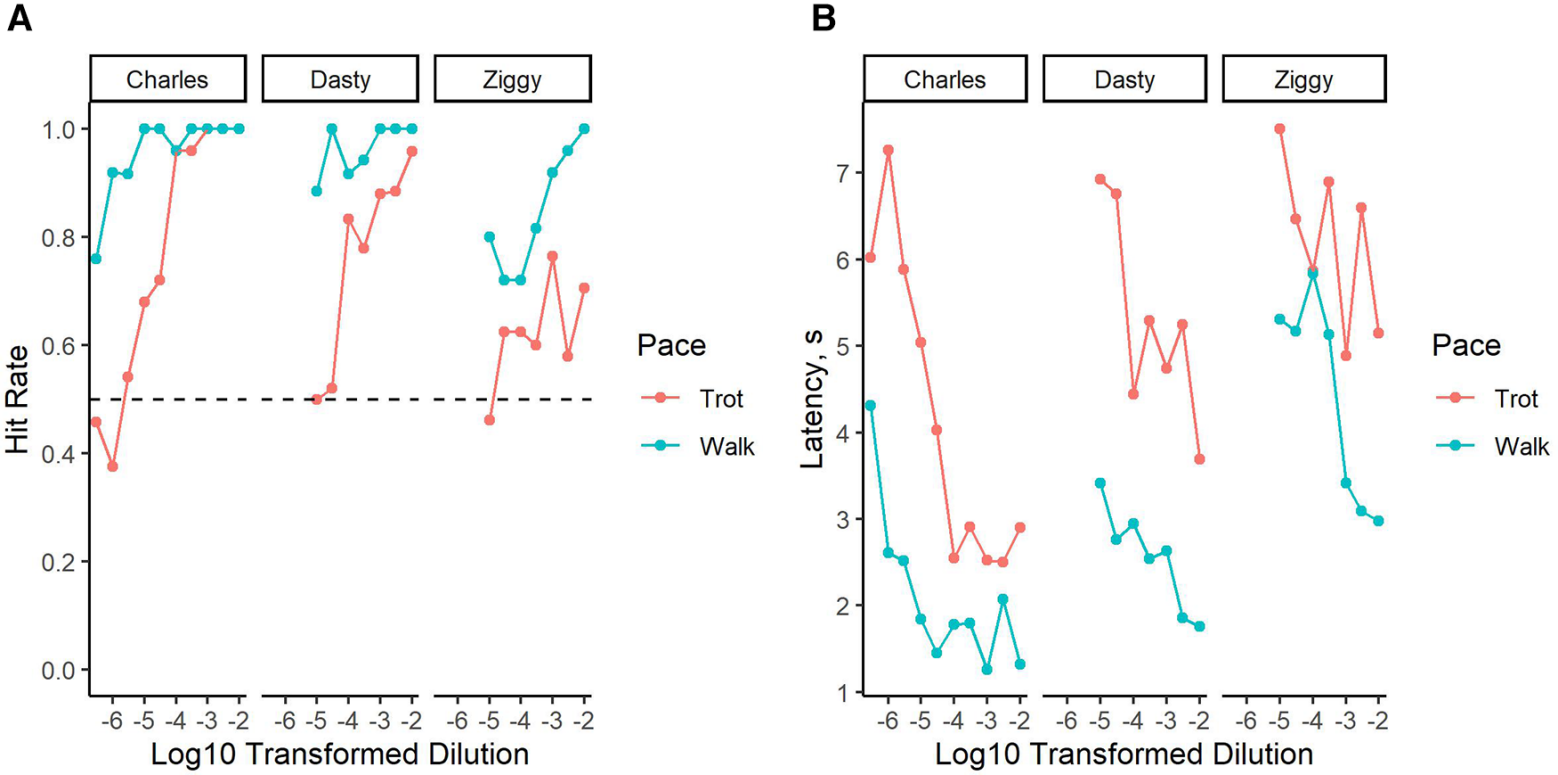
Performance decrement under physical exertion. (**A**) Dashed lined indicates a hit rate of 0.50, our threshold criterion. (**B**) Change in latency to respond on an odor present trial.

Dasty and Ziggy reached threshold at step 2 (8 testing sessions; 800 trials in total) and Charles reached threshold at step 3 (12 testing sessions; 1200 trials in total). All dogs reached detection threshold at the 8 km/h condition first while remaining above threshold at the same concentration at 4 km/h (see Figure 5A). Charles showed minimal impact of exercise intensity on hit rate at the high concentrations, with indistinguishable performance between the low and moderate intensities until 10^−4.5^ concentration at which point performance dropped precipitously at moderate compared to low intensity. Dasty showed a slightly poorer hit rate at higher concentrations at moderate intensity but with substantial separation between conditions at a concentration of 10^−3.5^ or lower. Ziggy showed substantial separation in conditions at all concentrations. This difference in Ziggy's performance could have been due to her previous inexperience in odor detection or normal variation between individuals. Interestingly, even at the higher concentrations all dogs took longer to respond to the target odor during moderate vs. low exercise intensity (Figure 5B). As expected, the time to indicate odor presence increased with decreasing concentration at both speeds.

**Figure 6:**
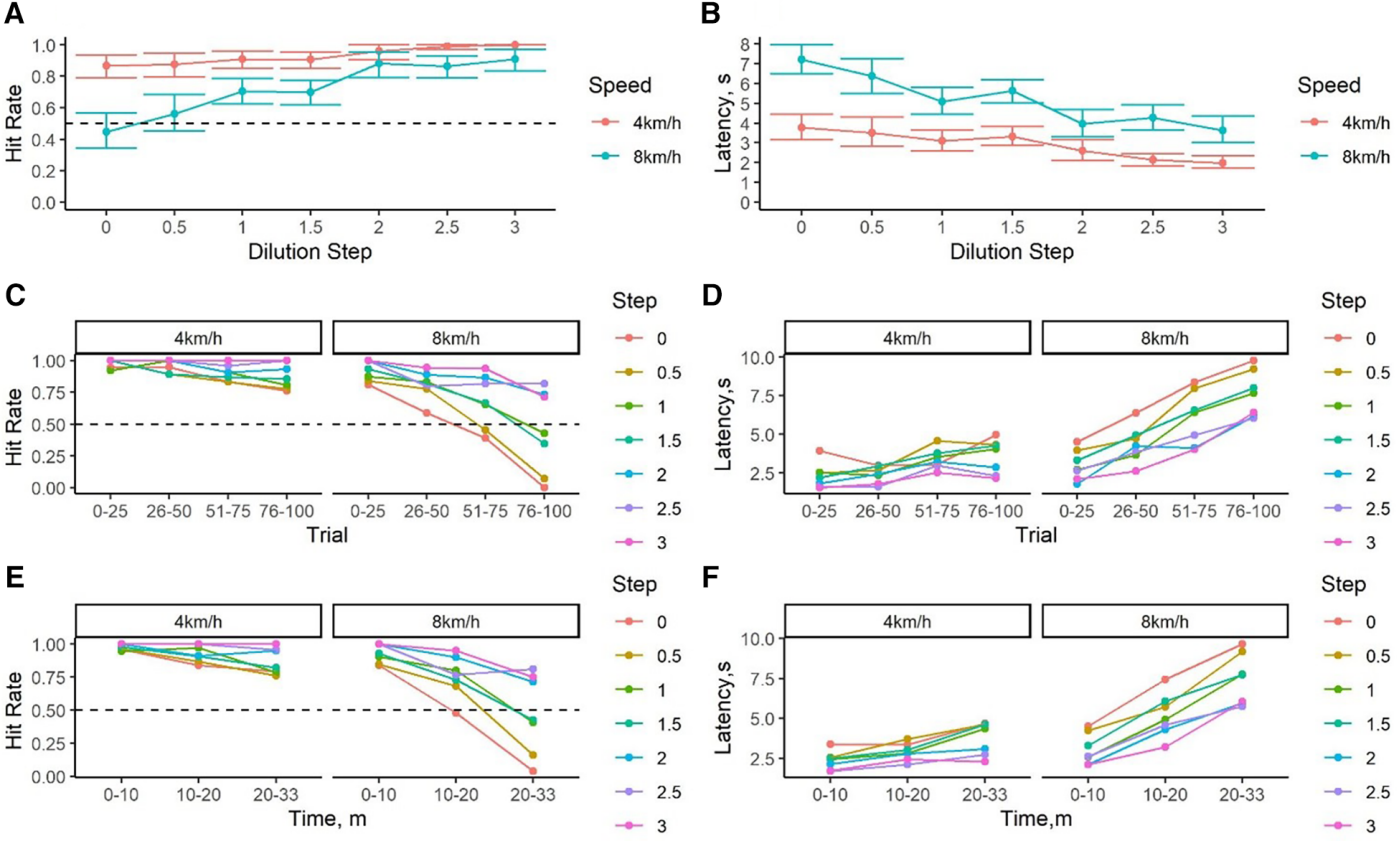
Performance decrement in reference to threshold and session duration. (**A**) Average hit rate ±95% confidence interval for the target odor at varying concentrations expressed as dilution steps. Dashed lines indicate a hit rate of 0.50, which was our threshold criterion. (**B**) Average latency to respond to the different concentrations tested expressed as dilution steps above threshold (**C**) Average hit rate to the different concentrations tested divided by 25 trial-blocks within a session. (**D**) Latency to respond across trials. (**E**) Average hit rate to the different concentrations tested by duration (min) of the session. Replots the data of (**C**), showing actual trial session time (exercise duration) on the *x*-axis. (**F**) Latency to respond across session duration (min). Replots the data of (**D**), showing actual trial session time (exercise duration) on the *x*-axis.

Figures 6A,B show the same data with concentration scaled to “dilution steps” (see Statistical Analysis). Even at the lowest dilution step (step 0; threshold) dogs' hit rate when exercised at 4 km/h (low intensity) remained high (0.87 ± 0.04). Hit rates to concentrations below 2 dilution steps (i.e., within 1–100× detection threshold) were lower at the moderate intensity compared to the low intensity sessions. From 2 to 3 dilution steps (100–1,000× detection threshold), there were minimal differences in hit rates between the exercise conditions. Interestingly, latency continued to show separation at all but one concentration, indicating that dogs required longer to respond to the odor independent of concentration when exercised at moderate intensity compared to low intensity.

Figures 6C,D show changes in performance with respect to trial number within a session. These results show that the hit rate at all concentrations started high for both exercise conditions in the first 25 trials. Hit rate declined across trials for dilution steps lower than 3, showing that independent of exercise intensity, dogs' hit rate for low concentration odors decreases with trial. Figures 6E,F show that this pattern is identical when considering the session time (exercise duration) compared to trial number. Most dramatically, the hit rate drops precipitously for the lowest dilution steps for the 8 km/h condition (moderate intensity), with no hits from trial 75 onwards [∼22.72 ± 0.28 (mean ± se) min after the beginning of the session, Figure 6E]. In contrast, dogs' hit rate to the lowest dilution step remained at 75% during the last 25 trials in a session at the low intensity exercise condition. At the same time, the hit rate for the highest concentration tested was ≥75% for both exercise intensities across all trials, indicating the effect of exercise intensity and duration was specific to more challenging odor concentrations. This is further exemplified in Figures 6E,F, which re-plots the *x*-axis with duration of the session. Within the first 10 min of exercise, performance is highly similar for both exercise intensities and all concentrations. By 10–20 min, the hit rate for the lowest concentration dropped to 50% at a moderate exercise intensity whereas it remained >75% at a low exercise intensity. By 20–30 min, dogs exercising under moderate intensity showed almost no alerts to the lowest concentration while hit rate remained at 75% during low intensity exercise.

**Figure 7:**
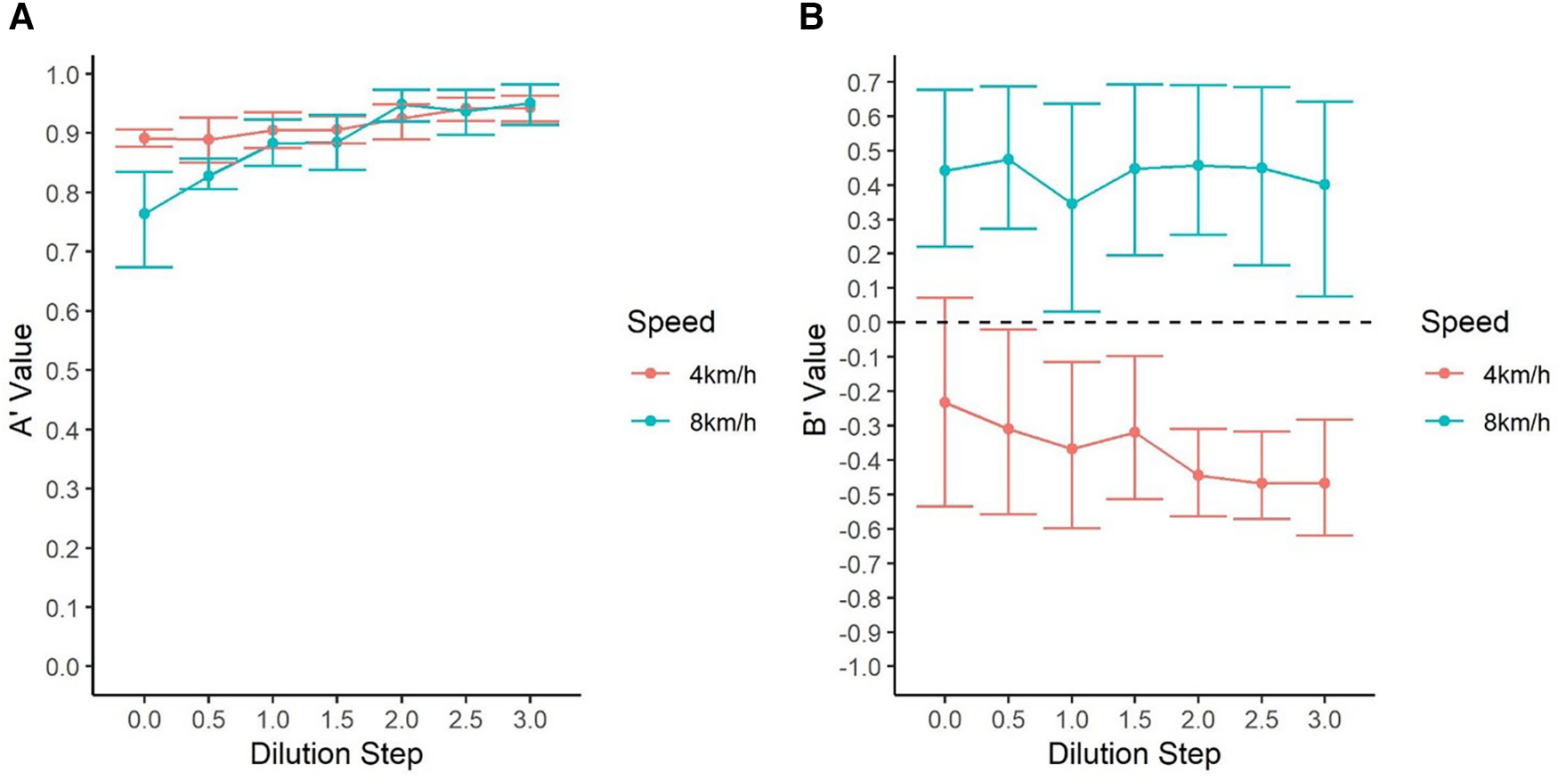
Signal detection analysis of dogs’ performance (**A**): dogs showed similar sensitivity (**A**’) to concentrations higher than 0.5 dilution steps independent of the speed at which they were exercising. Dogs’ sensitivity to threshold concentration (Step 0) was lower at 8 km/h (**B**): Shows the average (**B**”) values by concentration. All dogs showed a bias toward the go response at 4 km/h (higher false alerts) and a bias towards the No-go response at 8 k/m (more odor misses). Nonetheless, no difference in dogs’ bias was observed by concentration and it remained relatively constant depending on the speed at which they were exercising.

Signal detection measurements are illustrated in Figure 7. At 4 km/h (low intensity), *A'* values were >0.85 for all concentrations tested. *A'* dropped at moderate intensity when within 1 dilution step (10× threshold), indicating poorer discrimination between odor and controls. *B”* values show that dogs had a bias towards false alerting at low intensity exercise and a bias towards misses at moderate intensity. This bias remained consistent across all dilutions tested, indicating that the increase in misses at moderate intensity exercise were due to *A'* changes and not changes in bias.

## Discussion

Information provided by detection dogs is heavily relied on by the military, homeland security, and search and rescue. However, little research has been done to evaluate dog performance given the physical demands working dogs are faced with. This study sought to determine the effects of exercise duration and intensity on dogs' olfactory detection thresholds. Results suggest a negative relationship between exercise intensity and duration and odor detection threshold. During the first 10 min, dogs showed >80% hit rate for all the concentrations tested at both the low and moderate intensity exercise. After 20 min of moderate exercise intensity, hit rate for the lowest concentration dropped to 0% (100% misses) whereas the same dogs achieved >75% hit rate for the same concentration and duration under low intensity exercise. Conversely, dogs' odor detection performance to higher concentrations (100–1,000× detection threshold) was not affected by exercise intensity or duration, indicating that dogs could maintain performance under the moderate intensity exercise for the highest concentrations tested. Importantly, these results indicate that when faced with low odor concentrations, dogs showed a decrement in performance well below high intensity or maximal exertion or overt physical fatigue.

The signal detection analyses further show that dogs were generally more biased to false alert at low compared to moderate exercise intensity, but these values remained relatively stable across all concentrations. Thus, as dogs approached detection threshold, they did not show bias changes, suggesting that dogs did not just simply shift to a preference for making No-go responses due to exercise intensity. The *A'* results highlight that the decrement at lower concentrations at 8 km/h was due to *A'* (sensitivity) changes, indicating dogs were becoming poorer at perceiving the difference between odor present and odor absent trials. Taken together, this suggests that the decrement observed at 8 km/h for lower concentration odors was due to dogs showing a sensory decrement (i.e., *A'*; sensitivity) rather than simply becoming less likely to make an alert response due to exercise (i.e., *B’’*).

The observed reduction in olfactory sensitivity (i.e., *A'*) with increased exercise intensity has significant implications for detection dogs. Our results suggest that after being exposed to prolonged moderate exercise intensity, dogs' ability to detect near detection threshold concentrations declines even when supra-threshold detection remains good. Near threshold concentrations can appear in operational scenarios from concealment of targets or because dogs may be less sensitive to the particular target (8). This is particularly important for dogs tasked to search extensive areas for long periods of time (e.g., search and rescue and military working dogs). Our data here suggests that peri-threshold detection from dogs working at a moderate intensity might be substantially reduced after 20 min of moderate exercise intensity.

Our study, while conducted with a limited sample size of 3 dogs, aligns with prior findings indicating a decline in olfactory performance following exercise on a treadmill (5–7). There are two main differences between our study and previous ones: (1) prior studies exercised dogs before the olfactory task while we exercised dogs as they were simultaneously doing the olfactory task, (2) we evaluated changes across different odor concentrations measuring changes in detection limits. These differences allowed us to elucidate subtle effects.

Perhaps the most interesting effect observed herein is the exercise intensity by duration by odor concentration interaction. Performance decline was only substantially observed at the lower concentrations at moderate exercise intensity and only after more than 10 min of exercise. At higher odor concentrations, lower exercise intensity, or within less than 10 min of exercise, the hit rate was consistently above 75%. This indicates that an explanation to the results observed herein is more complex than simply saying that higher exercise intensity leads to performance decrement. This generates several possible hypotheses for future studies to better elucidate the observed effect.

For one, it is possible that a dog's reliance on mouth breathing for greater minute ventilation to exhale carbon dioxide or panting to dissipate heat during exercise may reduce sniffing. In a scenario where there is low odor concentration, every sniff may matter. Alternatively, this could be the result of dual task interference in which performing a motor task interferes with the ability to accurately perform a cognitive task. To our knowledge, dual task interference with odor detection has yet to be studied. In the experimental scenario here, the dog could not change their speed to focus on the odor because the motorized treadmill controlled the exercise speed. It would be interesting to repeat this experiment with a nonmotorized treadmill and see if odor accuracy remained high with high value rewards, would the dog's pace volitionally slow.

Further, this exercise duration and intensity interaction on detection sensitivity and response time suggests a possible competition for resources. There are numerous potential mechanisms which could include competition between the body vs. brain for fuel, blood flow and oxygenation, competition among working tissues needing oxygenated blood and fuel to metabolize for energy vs. cutaneous circulation needing to dissipate heat, or even competition between various neural networks needing to coordinate movement vs. those used for sensing and interpreting.

Lastly, although to the best of our knowledge exercise induced rhinorrhea (runner's nose) has not been studied in dogs, it could be a potential explanation for our results. Studies in humans have found that exercise induce nasal mucosal changes such as reduced ciliary beat frequency, prolong mucociliary transport, and increased neutrophilic infiltration (15). Furthermore, dehydration can cause the thickening of the nasal mucus and a reduction in mucus secretion. Although to our knowledge how all these changes in the nasal mucosa affect olfactory sensitivity has not been studied, it is possible that these changes in the nasal mucosa could have a negative impact on olfaction. These are all avenues of future research once a basic understanding of physiological differences at different exercise intensities and durations while detecting odor is established.

The Integrated Olfacto-Treadmill System is a novel tool that can be utilized to investigate the underlying physiological or cognitive cause of the observed decrement in olfactory threshold associated with increased exercise intensity. The controlled setup presented in our study offers an excellent platform to test different hypotheses and measure different parameters to develop models that can predict a decrement in olfactory sensitivity based on physiological (e.g., body temperature, heart rate, and respiration rate) measurements. Furthermore, by simply substituting the task to be performed, one could measure the effects of exercise on cognitive aspects and subsequently compare these results with those presented here to discern potential olfaction-specific effects.

## Limitations

There are several limitations to our research. First, we have a very small sample size. The requirements for a participant to be able to (1) walk and trot on a treadmill, (2) trot for ∼30 min, (3) learn an olfactory Go/no-go, and (4) complete the olfactory task while on the treadmill requires substantial training investment for privately owned dogs and limits the pool of dogs that can meet these requirements. This small sample size limited our ability to conduct null hypothesis testing. In addition to the small sample size, not all dogs had the same history or prior experience with exercise. For instance, prior to the study Charles was under a regular routine of strength conditioning. This might explain why his performance was better than the other dogs. Nonetheless, although he had a better threshold, he still showed the same decrement as the other dogs. Although further studies are needed to determine the cause of individual differences, the observed performance differences here are within the normal variation observed between individuals' threshold (16). Because Charles' and Ziggy's (dogs with the best and worst performance, respectively) HR at a trot were very similar, we suggest that differences in performance were most likely normal variation between individuals.

Second, we only used one target odor instead of a range of materials. Nonetheless, our conclusions are derived from the results obtained from 2,800 trials (over 800 trials per dog), our findings are consistent with previous research, and our data were consistent across dogs. Conducting further studies encompassing dogs from diverse backgrounds, breeds, and fitness levels are necessary to evaluate the generalizability of our results.

Third, we did not measure core body temperature, respiration, and other physiological parameters that can be used to further understand the underlying cause of the observed olfactory decrement. Fourth, the duration between each session was controlled by owner schedules and could be improved with a more systematic spacing of sessions. This would have ensured the same recovery time between session. Although due to the relatively low to moderate intensity of the exercise, this may not have been a significant factor affecting our results.

Fifth, we only tested dogs under two exercise intensities. Testing different permutations between exercise intensities within a session might have helped us better understand the underlying cause of the observed reduction in olfactory sensitivity. For example, would dogs' performance have recovered if we had reduced the exercise intensity after exercising for 20 min at moderate intensity?

Last, control of olfactory stimuli and concentration in a dynamic environment, such as while a dog is running on a treadmill, is inherently challenging and difficult to validate. We did show through a tracer molecule and photoionization detector, that there was relatively stable odor concentration across various locations (within a half-log step) on the treadmill; however, we had to use a tracer molecule and use concentrations greater than the dogs' detection limits. Thus, there remains a possibility of different concentrations across the treadmill and where the dog is located, which is an important future consideration. Nonetheless, the results obtained from this research provide valuable data that can guide future research in this area.

## Conclusions

Coupling the use of an olfactometer with a treadmill, we developed a paradigm where dogs can perform an olfactory task while exercising. Our findings reveal a discernible decrement in detection at peri-threshold concentrations after 20 min at moderate intensity exercise. Although further studies with more subjects are needed, these results provide an initial proof of concept that exercise intensity can negatively impact olfactory detection thresholds and that there is an interaction between exercise intensity and duration. The paradigm described here can be utilized to further quantify this effect.

## Data Availability

The original contributions presented in the study are included in the article/**Supplementary Material**, further inquiries can be directed to the corresponding authors
