# Supplementary material for: A canine model to evaluate the effect of exercise intensity and duration on olfactory detection limits: the running nose
Source: Front Allergy. 2024 May 9;5:1367669. doi: 10.3389/falgy.2024.1367669 (PMC11111909; doi:10.3389/falgy.2024.1367669)
Supplement: Supplementary file 1 [file Table1.docx]

**Descriptive information of dogs**

| Dog | Sex | Age | Breed | Size |
| --- | --- | --- | --- | --- |
| Charles | Neutered male | 3y | Mix | Medium |
| Dasty | Neutered male | 3y | Labrador | Medium |
| Ziggy | Spayed Female | 3y | Mix | Medium |
