# Supplementary material for: A canine model to evaluate the effect of exercise intensity and duration on olfactory detection limits: the running nose
Source: Front Allergy. 2024 May 9;5:1367669. doi: 10.3389/falgy.2024.1367669 (PMC11111909; doi:10.3389/falgy.2024.1367669)
Supplement: Supplementary file 3 [file Datasheet2.pdf]

Supplementary material captions

The raw data for this research is available in the supplementary materials. The Excel document includes 37 columns with all the data of this project. Below is a detailed explanation of how to navigate and understand the raw data.

1. subjID= The name of the dog.
2. expID= The experimenter running the session
3. session= The overall testing session number up until that point.
4. correctPort= Indicate if the trial contained the target odor (Olfactometer11) or if it was a blank trial (blank).
5. Response = Dogs response in the trial. All clear (no response; No-Go) Olfactometer 11 indicates dog responded to the stimulus presented (Go response).
6. Latency= Time to make a response. 10 indicates no response was recorded.
7. Poke1number= number of times the dog broke the IR beam.
8. Cumulative1= nose hold duration
9. port1Odor and Concentration= Concentration of the odor presented
10. TrialNumber= Trial within a session
11. Pace= The exercising pace within a session Walk = 4kmh and trot = 8kmh.
12. Trail= Trials grouped in blocks of 25 trials.
13. Correct= binomial variable to indicate if the response was correct (1) or incorrect (0)
14. FALSE_= binomial variable to indicate a false alert (1) or not (0)
15. Miss= binomial variable to indicate an odor miss (1) or not (0)
16. Alert= binomial variable to indicate if the dog responded within a trial (1) or not (0)
17. Axis1, Axis2,Axis3, and VM= Accelerometry data from the Actigraph.
18. Cum1,cum2,cum3,cumVM,= cumulative accelerometry values recorded up until that trial
19. TTtime= Trial duration in seconds.
20. Vs, CumulativeAccell= Dummy variables; not relevant
21. TTIMEcum= Cumulative time since the beginning of the session in seconds.
22. Trialminutes= Cumulative time since the beginning of the session in minutes.
23. HR and HR_AG= Average Heart rate within a trial.
